# 4.Q. Workshop: Showcasing PHIRI use case results measuring the impact of COVID-19 on population health

**DOI:** 10.1093/eurpub/ckac129.272

**Published:** 2022-10-25

**Authors:** Organised by: PHIRI, EUPHA-PHMR, Chair persons: Enrique Bernal-Delgado (Spain), Thomas Ziese (Germany)

## Abstract

The COVID-19 pandemic has clearly demonstrated the urgent need for a cross-border and structured European mechanism to exchange, organise and access reliable health information between countries, especially in the area of population health. Population health information, defined by data on health status, health determinants and healthcare systems performance, allows for oriented research to increase the knowledge base in Europe and underpin political decision-making. Its exchange requires timely and topical provision of high-quality health information. There are many indirect effects of the COVID-19 pandemic that affect health through various pathways including secondary consequences on health and wellbeing due to delayed prevention, diagnosis and medical treatment. Within the Population Health Information Research Infrastructure (PHIRI) we look at four use cases measuring the impact of COVID-19 on population health and demonstrating how a broad variety of routine data can be pooled and/or used for secondary analysis in a distributed way across Europe aiming to facilitate research by making scalable, reproducible methods available. These use cases represent pilot activities for the benefits and added value of an infrastructure supporting federated analysis by bringing together data from different European countries and feeding the results into the federated research infrastructure. In over 20 data hubs, data is mobilised and ready to be analysed in a distributed manner. The use case outputs will be processed in an interoperable way by formalising data models, data management processes and analytical pipelines, all of which are part of the client-server PHIRI federated infrastructure implemented as here 10.5281/zenodo.6483177. The workshop aims to ensure a better understanding of COVID-19 impacts in specific subgroups and risk settings by conducting research through real-life use cases of immediate relevance. The FAIRified use cases analysis results focusing on comparisons between countries are presented and provide actionable outcomes to guide policy makers in preparedness and response scenarios. Knowledge and expertise developed across Europe is shared in this workshop. The four presentations will focus on selected aspects of COVID-19 impacts on population health. The first presentation will be on direct and indirect determinants of COVID-19 infection and outcomes in vulnerable population groups with reference to inequalities. This will be followed by a contribution of COVID-19 related delayed care in breast cancer patients. The third presentation looks at the impacts of COVID-19 on perinatal health inequalities followed by the fourth on insights in COVID-19 related changes in population mental health. Exchange with the audience will facilitate knowledge and opinion exchange through an interactive Mentimeter poll during the session.

**Key messages:**

• The results will support the exchange of knowledge and expertise by facilitating insights in the impacts of COVID-19 in specific subgroups and risk settings compared across European countries.

• Actionable outcomes to guide political decision-making in preparedness and response scenarios will be provided.

